# Bridging the gap: evaluation of the impact of a structured pre-professional medical education gap year on medical career pathway, competency development, and preparedness for professional school

**DOI:** 10.1186/s12909-025-08501-z

**Published:** 2026-01-17

**Authors:** R. Maxwell Regester, Amy C. Cannella, Alan R. Erickson, Harlan Sayles, Amy Rau, Anthony J. Griess

**Affiliations:** 1 College of Medicine, University of Nebraska Medical Center, Omaha, NE USA; 2 Dermatology Specialists of Omaha, Omaha, NE USA

**Keywords:** Gap year, Career exploration, Competency development, Professional preparedness, Burnout prevention

## Abstract

**Background:**

Medical school admissions committees often prioritize traditional pathways, but structured clinical gap year (scGY) programs may offer valuable opportunities for career exploration, competency development, and professional preparedness. The impact of scGY programs on these outcomes remains unclear. The objective of this study was to evaluate the impact of a structured clinical gap year program on medical career exploration, competency-based skill development, preparedness for professional school, and burnout mitigation.

**Methods:**

This observational study surveyed 38 participants who completed a 14-month dermatology-focused scGY program at a single private practice. The scGY program began in 2017 and remains ongoing. Surveys were administered in May 2024, with all responses submitted by July 2024. The program included direct patient care, surgical assisting, electronic medical record use, structured mentorship, and weekly didactic sessions. Thirty-seven participants responded to a post-program survey (97% response rate). Data collected included academic outcomes, self-reported confidence in ACGME core competencies (before and after the program), perceived preparedness for professional school, and perceptions of whether a gap year helps mitigate burnout. Changes in competency confidence were assessed using Wilcoxon signed-rank tests.

**Results:**

Participants reported significant improvements in all competency domains, with the greatest increases in patient care/clinical skills (mean improvement: 6.2 points) and interpersonal/communication skills (mean improvement: 5.9 points). Compared to their peers, 64% self-reported they felt more prepared and 36% self-reported they felt much more prepared for professional school. Career exploration rankings indicated the scGY program was more valuable than shadowing or volunteering. Additionally, 89% believed that completing a scGY reduced burnout.

**Conclusions:**

A structured clinical gap year program enhances perceived competency development, affirms career pathways, and improves professional school self-reported preparedness while potentially reducing burnout. These findings suggest that admissions committees should recognize scGY experiences as valuable, rather than viewing them as unnecessary delays in medical training.

**Supplementary Information:**

The online version contains supplementary material available at 10.1186/s12909-025-08501-z.

## Background

Amongst medical school admissions committees, pre-medical advisors and even students, there is uncertainty about the best pathway to medical school. Some believe the traditional path – direct matriculation to professional school after completing an undergraduate degree – is the most effective route to becoming a successful health professional [1]. This view often casts alternative pathways, such as paid healthcare experiences (gap years), as unnecessary delays or even as potentially detrimental interruptions in a student’s progression toward a medical career [1]. However, emerging evidence suggests that these non-traditional routes, including structured clinical gap year (scGY) experiences, may offer unique opportunities for growth, career exploration, and competency development [2–4].

This discussion is particularly relevant in today's competitive landscape. The American Association of Medical Colleges (AAMC) reports that for the academic year 2023–2024, a staggering 52,577 individuals submitted nearly one million applications to MD-granting medical schools. Despite the average applicant applying to 18 schools, only 43.7% were accepted, showcasing the intense competition in the field [5]. The competition becomes even stiffer when applying to more selective schools, with acceptance rates as low as 1.2% [6].

On the American Medical College Application Service (AMCAS) application, students submit undergraduate coursework, grade point averages (GPA), Medical College Admission Test (MCAT) scores, personal statements, and letters of recommendation. An additional section allows applicants to share work experience, extracurricular activities, awards, honors, and publications, with the opportunity to highlight three that were most meaningful [7]. While all elements of the application are perceived as important, the relative weighting of each factor in acceptance or rejection by admissions committees is not publicly disclosed and remains unclear to applicants.

There are many pathways taken prior to medical school. Three of the most common include: 1) immediate matriculation after a four-year degree program (traditional), 2) non-traditional students matriculating after different life experiences, and 3) students who, by choice or circumstance, take a gap year between college and professional school. Regardless of the path, all applicants try to determine which activities will bolster their application for admission.

Admission committees operate confidentially and do not make their internal processes widely available. Considerable variation likely exists between institutions and among members of an individual committee who may weigh elements of the application differently. They aim to select students who demonstrate the cognitive traits and personal characteristics requisite to the practice of medicine [10]. Ideally, applicants should also demonstrate adequate career exploration to affirm their commitment to the grueling pathway of the profession.

Studies show that objective measures, such as the MCAT score and, to a lesser degree, the undergraduate GPA [8], can reliably predict short term medical student success. This predictive ability has not been seen in more subjective assessments, such as interview scores and holistic review [9]. Objective metrics speak to academic ability, and the subjective letters of recommendation and interview scores may identify desired personality traits. However, less easily discerned are the intangible attributes of career exploration, including a commitment to patient care and requisite professionalism, communication skills, and dedication to self-improvement [9, 10].

Most applicants aim to demonstrate career exploration via their portfolio of extracurricular activities. In a 2023 survey by the AAMC, medical school admissions officers ranked the experiences of medical and non-medical volunteerism, clinical shadowing and leadership of higher importance than paid healthcare employment when making decisions about who to interview and accept [10].

Each admission committee may view the portfolio of experiences differently, making their intended outcomes poorly defined and difficult to measure. Furthermore, applicants participate in diverse individualized experiences, making direct comparisons a challenge. Although important pieces of the application puzzle, limited data, if any, exists about which experiences predict success in medical school and beyond.

One recent study of prior healthcare employment (PHE) showed that 49 out of 434 students who participated in a minimum of six months of any type of PHE before medical school outperformed their peers in the clinical training environment. The authors propose that this early contextual learning enhances patient care and documentation skills, strengthens teamwork, feedback, and communication, and reflects personal traits like work ethic, motivation, and resilience [2].

While PHE shows improved outcomes, it ranks lower than other experiences by admission officers. Pre-medical advisors are uncertain how to guide their advisees who desire to take a gap year in health care employment. To improve advising practices, pre-medical advisors have called for the study of this group of students with respect to the utility of a gap year on performance and the perception of the experience by students and medical professionals [11].

This study evaluates a structured clinical gap year program (scGY) launched in 2017 for students aiming to attend medical school, then expanded to include other healthcare professional schools, including PA and Dental school. Applicants undergo interviews before joining clinical teams of physicians and physician assistants, where they participate in patient care, surgical assisting, and electronic medical record tasks as paid team members. The program is complemented by weekly lectures on high-yield topics, mentorship, research opportunities, and personalized recommendation letters. *A detailed description of the program is available in the supplemental information.*

With the uncertainty among pre-medical students, advisors and admissions officers about the utility of a gap year program, these investigators aim to determine if a pre-medical scGY experience can serve as adequate career exploration, show longitudinal improvement in competency-based domains and prepare students for success in professional school.

## Method

This study was deemed exempt by the University of Nebraska Institutional Review Board. Thirty-eight participants who completed a gap year program at a private dermatology practice in Omaha, NE, were contacted via email to complete a 44-question survey. This survey was conducted via REDCap, and statistical analysis was performed using STATA. Data was collected from 5/2024–6/2024. Metrics were collected on undergraduate school and major, GPA, MCAT scores, USMLE scores, chosen profession, and matriculation into professional school.

Twenty-eight competency-based questions were developed and grouped into categories using three Midwest medical schools’ published Program Objectives, utilizing domains in alignment with the Accreditation Council for Graduate Medical Education (ACGME) framework. The competency domains (number of questions) included medical knowledge (4), patient care and clinical skills (4), practice-based learning and improvement (2), interpersonal and communication skills (6), professionalism (4), systems of health care (3), interprofessional collaboration (2) and personal and professional development (3). Using a 10-point Likert Scale, where zero represents no knowledge or ability to perform the skill and 10 represents complete mastery of the subject or skill, respondents were queried about their confidence at the beginning and upon completion of the gap year.

Participants were also asked about participation in career exploratory experiences, preparedness and burnout perception compared to peers who did not complete a scGY. Data was collected on research activity. Open-ended questions were included to allow individualized comments. Information was also collected for programmatic improvement. *The full survey is available in the supplemental information.*

Data was summarized using descriptive summary statistics (counts and percentages) for all survey items. Wilcoxon signed-rank tests were used to evaluate changes in ratings from pre- to post-gap year for each competency-based question. Open-ended questions were thematically analyzed. Not all participants responded to every open-ended question.

## Results

Of 38 gap year participants—from those who joined the program starting in 2017 through May 2024 when data were collected—37 returned surveys (response rate of 97%). One of the 37 respondents was also an investigator. To minimize bias, only objective descriptive characteristics (e.g., age, academic background) were included for this individual, and their competency self-assessment data were excluded, as their dual role may be a conflict of interest. Thus, competency questions were analyzed for 36 of 37 surveys (97%), and participant descriptors were analyzed for 37 of 37 surveys (100%). These participant descriptors are summarized in Table 1, highlighting the diverse geographic and professional background of the scGY participants.

**Table 1:** Descriptive characteristics of structured clinical gap year participants

	Number
Total Number of Participants	37
Gender	
Male	12
Female	25
Number of Undergraduate Institutions Represented	14
Number of Undergraduate Cities Represented	12
Number of Undergraduate States Represented	8
Undergraduate Majors	
Biology	14
Biochemistry	7
Other Science Majors	12
Non-Science Majors	4
Undergraduate Mean GPA	3.8 (range 3.3—4.0)
Mean MCAT Score	510 (range 495–525)
Applied to Professional School Prior to Gap Year	
Yes	17
No	20
Accepted or Matriculated into Professional School	
Yes	30
No	7
Professional School Attended after Gap Year	
MD	16
DO	6
PA	5
Master of Medical Science	1
Master of Public Health	1
Post-Baccalaureate Program	1

Professional School Attended after Gap Year is only known for the 30 scGY participants who have matriculated into professional school

### Career exploration

Of all scGY participants, 13 (36%) were more likely and 18 (50%) were much more likely to pursue a career in healthcare after completing the program. One participant (3%) was much less likely to pursue a career in healthcare.

Thematic exploration of open-ended questions regarding the impact of the scGY experience on a participant’s acceptance into professional school revealed 17 (49%) perceived a positive overall impact and 7 (20.0%) perceived an enhanced interview performance. The most common specific impacts on professional development were increased clinical experience (18; 51%) and increased real-life understanding and application of medical/patient care (14; 40%).

**Fig. 1:**
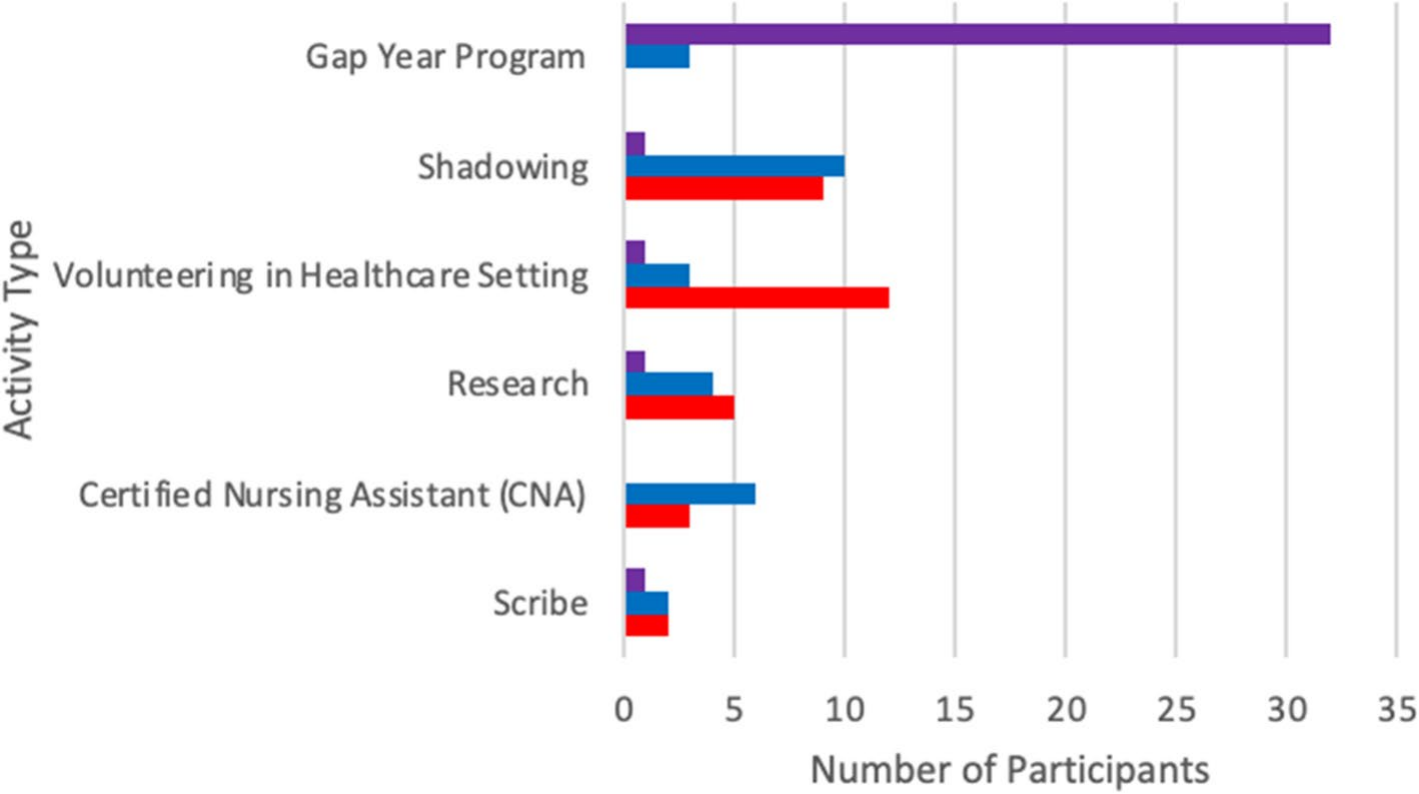
Represents the scGY participants’ rankings of exploratory experiences preparation to succeed in the pre-clinical years of training. Purple represents the 1st most, blue the 2nd most and red the 3rd most important

In addition to the scGY Program, participants had engaged in multiple additional career exploratory experiences including 36 in shadowing (100%), 31 volunteering in health care (86%), and 23 participating in research (64%). Among all activities, the scGY Program was ranked the most important for preparation to succeed in the pre-clinical years of training, with 32/35 (91%) participants listing it as the most important. No other activity received more than one vote for the most important. Shadowing was ranked the second-most important and volunteering in a health care setting was ranked the third-most important (Fig. 1).

Participants were asked to rank the importance of shadowing for career exploration on a 0–10 scale, and the overall average was 6.3. Thematic analysis of individualized comments about shadowing likes and dislikes revealed both positive and negative remarks (12), only negative remarks (9) and only positive remarks (6). The most common positive themes included exposure to different specialties (10; 35%) and gaining insight into the daily life and responsibilities of a provider (9; 31%). The most common negative themes were not being hands-on/lack of active participation (9; 31%) and that shadowing does not fully/accurately represent the profession (6; 21%).

### Competency domains

**Fig. 2:**
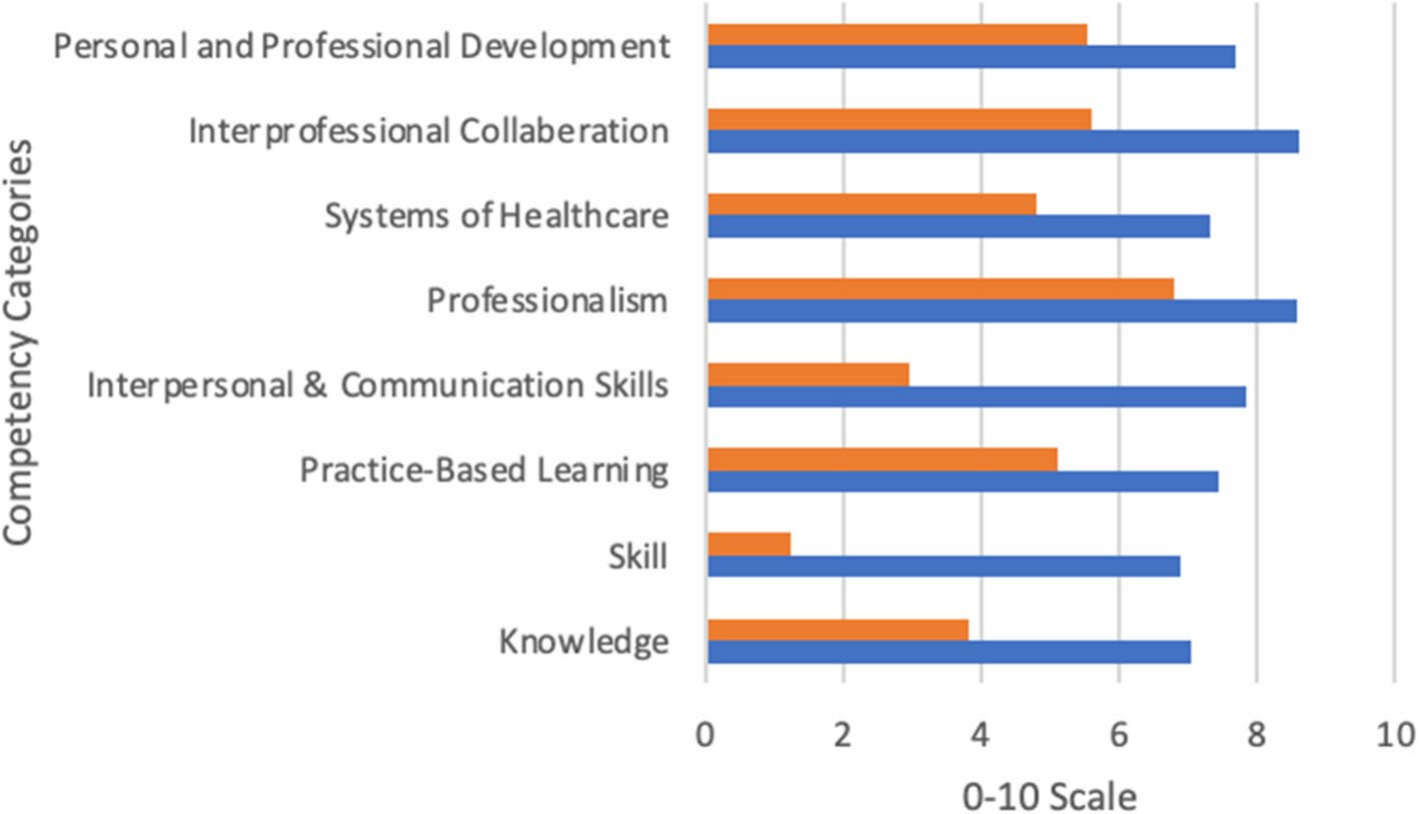
Shows average points scored across each competency domain at the start (prior, orange bars) and upon completion (post, blue bars) of the structured clinical gap year experience. Zero represents no knowledge or ability to perform the skill and 10 represents complete mastery of the subject or skill

Using Wilcoxon signed-rank tests, all 28 questions in eight competency domains showed significant improvement from pre- to post-gap year training (p < 0.001 for all tests). The average increase in competency across all questions was 3.5 out of 10 points (range 1.3 to 6.4 points). Figure 2 shows question means grouped by domain. The largest overall point improvements were seen in the domains of patient care and clinical skills: obtain patient history (from 2.1 to 8.3) and perform patient care (from 1.3 to 7.6), and interpersonal and communication skills: document in EHR (from 1.9 to 8.3) and present relevant patient information (from 1.8 to 7.8).

### Preparedness

**Fig. 3:**
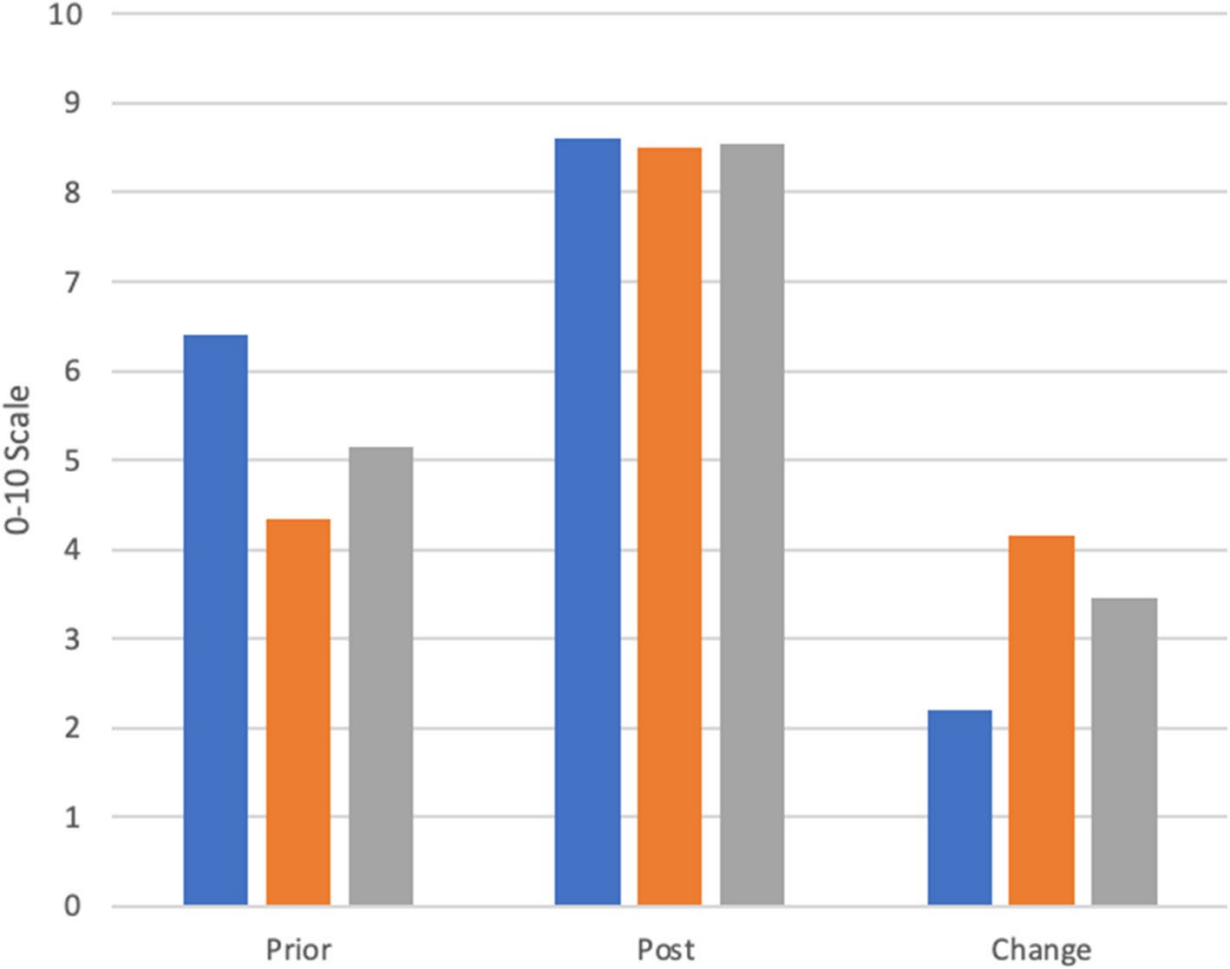
Shows scGY participants’ ratings of overall preparedness for the next professional step and working in a professional setting compared to their peers who did not take a similar gap year. Zero represents being not at all prepared and 10 represents being completely prepared. Blue represents Group 1, orange Group 2, and grey all participants

Participants were sorted into two groups. Group 1 consisted of those in the last month of the program and Group 2 consisted of those who completed the program at least one year prior. This stratification was intended to capture potential differences in perceived preparedness, particularly among those in Group 2, who had already experienced graduate or professional school and could more accurately assess how well the program prepared them relative to their peers. Participants rated their preparedness for their next professional step before and after the program on a 10-point Likert scale. (Fig. 3) Overall, participants felt more prepared for their next academic or professional step (increase of 3.4) and more prepared to work in a professional setting (increase of 3.0). Of the 22 participants who matriculated into professional school, 14 (64%) felt more and 8 (36%) felt much more prepared to succeed compared to their peers who did not complete a scGY. Of all scGY participants, 18 (51%) felt more and 15 participants (43%) felt much more prepared. Of the scGY participants, all who had taken USMLE Step 1 (*n* = 9), all who had taken Step 2 (*n* = 6), and the single participant who had taken Step 3 passed on their first attempt.

### Burnout

Thirty-two participants (89%) agreed with the statement that students who complete a gap year have lower rates of burnout when attending professional school. Thematic analysis of comments revealed the scGY provided motivation to continue schooling/pursuit of a medical career (6 participants, 38%) and it gave time and opportunity to pursue interests outside of school (5 participants, 31%).

3.5 Research Activity.

Seven (18%) scGY students participated in scGY-faculty supported research, including protocol development, IRB submission, data collection and abstract or manuscript preparation that has or will result in presentations or publications.

## Discussion and conclusions

This study shows that participating in a robust clinical health care employment experience prior to starting professional school can be a meaningful experience and should not be broadly assumed to reflect delayed academic readiness.

This is the first study to our knowledge that demonstrates that students rate a scGY as superior to other career exploratory activities, although this finding should be interpreted cautiously given the lack of a control group and the potential for selection and confirmation bias. Participants noted that the program provided a realistic perspective of the medical profession and the hands-on experience that they crave, helping them solidify their decision to pursue a career in healthcare. Ranked second in our study, shadowing is perceived by many as the gold standard for career exploration [10, 11]. Our analysis revealed significant limitations in shadowing, including limited hands-on experience, poor engagement and lack of access. Though admission committees value volunteering and students validated its importance, it was ranked third in exploratory importance. Our study suggests a disconnect between the importance ratings of experiences in an application portfolio between admissions officers and the applicants [10].

Participation in the scGY program led some to the realization that healthcare was not the right career path, while others changed their trajectory within the health care field. All participants rated their experience in the program as positive, suggesting these changes were not the result of poor mentorship or a negative program experience, but rather robust career exploration. Recognizing that a chosen career path may not be the right fit prior to enrolling in professional school may represent one of the most valuable outcomes of the scGY program.

Every participant in our study who completed the scGY Program and enrolled in medical professional school felt more prepared to succeed compared to their peers who did not complete a similar gap year. That said, this is purely self-reported data without a control group, so the statistical significance of this finding is not certain. This finding is in alignment with a previously published study in the United Kingdom that found that 77 of 79 gap year participants and 48 of 105 non-gap year participants who subsequently enrolled in medical school supported taking a pre-medical gap year [3]. As many medical schools are moving clinical training earlier in the curriculum, students are asked to simultaneously adapt to the academic rigor and perform in the high-stakes clinical environment of medical school. This poses a significant challenge to most students, and having a year of clinical professional training and identity formation can mitigate many of the clinical environment stressors.

A study looking at the effects of early clinical exposure by certifying as an emergency medical responder before starting coursework in the first year of medical school showed improved subjective reporting of competencies. When investigators looked further, the greatest improvements were seen in students who also had additional PHE [4]. Our competency outcomes endorse this finding, showing significant improvements in every question of each domain. We saw the greatest improvements in patient care and clinical skills and interpersonal and communication skills. These subjectively reported outcomes should be further studied to determine if PHE can effectively “prime the pump,” allowing more rapid incorporation of knowledge, skills and attitudes into a solid foundation of prior contextual learning.

A key advantage of the scGY Program is the mentorship component. Participants highlighted the value of personalized guidance from practicing physicians, which aided in career decisions and strengthened applications with impactful letters of recommendation. The program also fostered peer support, enhancing both personal and professional development, and one participant stated, "I really enjoyed having other gap years my age who were also going through the application process because it gave me a support system.” Because poor family and social support have been shown to be risk factors for medical student suicidal ideation [12], future studies should evaluate the impact of pre-matriculation mentorship and well-developed peer support systems on subsequent mental health challenges.

In the context of the growing epidemic of burnout among physicians and medical trainees, a previously published study demonstrated that students who took a gap year prior to entering a combined baccalaureate-MD program reported significantly lower levels of burnout [13]. Although our scGY participants were not assessed using validated burnout instruments, 89% agreed that a gap year program reduced burnout during professional school. The potential benefits may relate to increased time for self-reflection, engagement in non-medical interests, enhanced preparedness for academic demands, and the development of support networks. These findings merit further investigation.

The opportunity to participate in research is also a positive outcome of the scGY program, with seven students producing work suitable for presentation or publication. Published data on non-clinical research gap year programs are at the pre-medical and post-graduate level. Reported benefits of these programs include increased number of publications, perceived application advantage for competitive programs, and off-loading clinical faculty with research demands for rank advancement [14–16]. The scGY program gives participants the simultaneous benefits of clinical training with opportunities to bolster their research portfolio. In turn, the clinical providers overseeing the students are afforded an engaged and motivated workforce, as well as opportunities to teach and mentor outside of a career in academic medicine.

This study has several strengths. First, is a high survey response rate, enabling the capture of nearly complete data. In addition, while most published data on gap year programs capture varying experiences and duration, this study evaluated a standardized program experience and duration.

This study has several important limitations, including a small sample size and the lack of a control group. Data was collected in a self-recall survey, introducing the possibility of recall and reporting bias. The survey instrument was developed with the assistance of a statistician but was not formally validated. Furthermore, participants were surveyed at the completion of the program, introducing potential bias in their self-perceived competency gains and reported levels of preparedness, as described by the Hawthorne effect. Generalizability may be impacted because all participants worked in a multi-specialty dermatology clinic, which limited exposure to other areas of medicine. However, only two of the scGY participants have pursued dermatology. Importantly, the clinical experience is essentially unchanged since the program inception in 2017, but the full program has improved over time to include a more robust didactic program and facilitated mentoring, making the positive findings more conservative.

Future research should concentrate on the long-term impacts of scGY programs on medical school performance, residency placement and overall career satisfaction. Longitudinal studies are needed to confirm whether the observed early benefits lead to sustained professional success and to develop scGY models that optimize educational outcomes. Additional research should also focus on scGY opportunities as an avenue for students under-represented in medicine to gain early access to clinical exposure and mentoring. Given our findings, which suggest that a scGY program may reduce student burnout, additional studies should explore this in greater depth, including the overall mental health impact and the experience of mentors who oversee a scGY program.

In conclusion, a scGY Program can offer significant advantages in both career exploration and perceived preparation for professional healthcare education, challenging the notion that the direct path from college to medical school is superior or preferred. A scGY program significantly develops self-reported confidence in core competencies essential for professional school and medical practice. Mentoring, research opportunities, peer support networks and reduced burnout are additional perceived benefits of the program. Far from being a fallback option, a scGY allows students to affirm their career choice, develop key skills, and potentially enter professional school better prepared for success. Admission committees should recognize these programs as a legitimate and valuable pathway to the medical profession, dispelling the bias that such experiences signal a lesser applicant.

## Supplementary Information

Supplementary Material 1.

Supplementary Material 2.

## Data Availability

Final survey and description of the structured clinical gap year program included in supplementary information files. Collected survey data available upon request.
