# Supplementary material for: Bridging the gap: evaluation of the impact of a structured pre-professional medical education gap year on medical career pathway, competency development, and preparedness for professional school
Source: BMC Med Educ. 2026 Jan 17;26:248. doi: 10.1186/s12909-025-08501-z (PMC12895797; doi:10.1186/s12909-025-08501-z)
Supplement: Supplementary file 1 — Supplementary Material 1. [file 12909_2025_8501_MOESM1_ESM.docx]

**Dermatology Specialists of Omaha Gap Year Survey**

Completion of this survey implies consent to use de-identified data in aggregate to improve the Gap Year Program and assist future Gap Year students to matriculate into their chosen profession.

**Competencies**

On a scale of 0-10 (with “0” being no knowledge and “10” being complete mastery of the subject), please rate your level of **knowledge** both prior to AND after your gap year for each of the following:

1. Overall Basic Science Knowledge
   1. Prior to gap year:
   2. After your gap year:
2. Anatomy Knowledge
   1. Prior to gap year:
   2. After your gap year:
3. Overall Clinical Science Knowledge
   1. Prior to gap year:
   2. After your gap year:
4. Dermatology Knowledge
   1. Prior to gap year:
   2. After your gap year:

On a scale of 0-10 (with “0” being no ability to perform the skill and “10” being complete mastery of the skill), please rate your **level of ability** to perform the following tasks both prior to AND after your gap year:

Patient Care and Clinical Skills

1. Obtain a patient history.
   1. Prior to gap year:
   2. After your gap year:
2. Perform a targeted examination.
   1. Prior to gap year:
   2. After your gap year:
3. Perform patient care skills (suturing, doffing/donning, sterile technique, surgical assistance, bandaging, wound care)
   1. Prior to gap year:
   2. After your gap year:
4. Interpret pathology reports.
   1. Prior to gap year:
   2. After your gap year:

Practice-Based Learning and Improvement

1. Identify and resolve gaps in knowledge using self-directed learning.
   1. Prior to gap year:
   2. After your gap year:
2. Accept and integrate formative feedback.
   1. Prior to gap year:
   2. After your gap year:

Interpersonal & Communication Skills

1. Use appropriate medical terminology.
   1. Prior to gap year:
   2. After your gap year:
2. Effectively communicate with patients and family members
   1. Prior to gap year:
   2. After your gap year:
3. Relay difficult news to patients in an empathetic and caring manner.
   1. Prior to gap year:
   2. After your gap year:
4. Present relevant patient information to a provider/attending physician.
   1. Prior to gap year:
   2. After your gap year:
5. Effectively communicate with members of the health care team
   1. Prior to gap year:
   2. After your gap year:
6. Document in the electronic health record (EHR)
   1. Prior to gap year:
   2. After your gap year:

Professionalism

1. Demonstrate accountability, reliability, and responsibility.
   1. Prior to gap year:
   2. After your gap year:
2. Demonstrate sensitivity to patient needs based on individual differences and background.
   1. Prior to gap year:
   2. After your gap year:
3. Identify the importance of and show compliance with HIPAA.
   1. Prior to gap year:
   2. After your gap year:
4. Act in accordance with a professional environment.
   1. Prior to gap year:
   2. After your gap year:

Systems of Health Care

1. Identify patient barriers to care.
   1. Prior to gap year:
   2. After your gap year:
2. Account for patient safety.
   1. Prior to gap year:
   2. After your gap year:
3. Advocate for patients struggling in the health care system.
   1. Prior to gap year:
   2. After your gap year:

Interprofessional Collaboration

1. Work as Part of a Medical Team
   1. Prior to gap year:
   2. After your gap year:
2. Foster a culture of teamwork.
   1. Prior to gap year:
   2. After your gap year:

Personal & Professional Development

1. Develop skills to sustain lifelong professional growth.
   1. Prior to gap year:
   2. After your gap year:
2. Evaluate and address personal limitations.
   1. Prior to gap year:
   2. After your gap year:
3. Identify tools for conflict management.
   1. Prior to gap year:
   2. After your gap year:

**Preparedness**

On a scale of 0-10 (with “0” being not at all prepared and “10” being completely prepared), please rate your level of **preparedness** both before AND after your gap year or the following:

1. Next Academic or Professional Step
2. Prior to gap year:
3. After your gap year:
4. Working in a Professional Setting
   1. Prior to gap year:
   2. After your gap year:
5. Compared to your peers who did not complete a gap year, how prepared did you feel to succeed in professional school?

| Much less prepared | More prepared | Neither more nor less prepared | More prepared | Much more prepared |
| --- | --- | --- | --- | --- |

**Exploratory Experience**

1. In which of the following activities did you participate **AND** list on your application to professional school?
   1. Certified Nursing Assistant (CNA)
   2. Emergency Medical Technician (EMT)
   3. Gap Year at DSO
   4. Laboratory Technician
   5. Medical Technician
   6. Phlebotomist
   7. Research
   8. Scribe
   9. Shadowing (not as part of the Gap Year)
   10. Volunteering in health care setting
   11. Other ____________________
2. Drop down box with the items selected above:

Please rank your top 3 activities in order of most to least important for preparation to succeed in the pre-clinical years of your training (you can only rank 3).

1. Please rank the importance of a traditional shadowing experience eon your exploration for a career in the medical profession: (0 = not important at all and 10 = extremely important).

What did you like or dislike about shadowing?

**Miscellaneous**

1. Following the gap year program, how much more or less likely were you to pursue a career in healthcare?

| Much less likely | Less likely | Neither more nor less likely | More likely | Much more likely |
| --- | --- | --- | --- | --- |

**Descriptive and Program Improvement Questions**

1. With respect to your undergraduate education
   1. Where did you attend college?
   2. What was your major/s?
   3. What was your GPA at graduation?
2. What was your highest MCAT score?
3. Did you apply to professional school prior to your gap year?
   1. If yes, what was the reason(s) you were not accepted?
      1. Low GPA
      2. Low MCAT
      3. Lack of career exploration
      4. Unknown
      5. Other ___________________
   2. If no, why did you select a gap year?
4. Were you or have you been accepted into professional school? (If yes, drop down)
   1. What is your chosen profession?
   2. What is the name of your professional school?
5. What role did the gap year program play in your acceptance to professional school?
6. Have you taken a USMLE Board Exam? (If yes, drop down the following/please put prefer not to answer)
   1. Did you pass Step I?
      1. How many attempts did it take you to pass?
   2. Did you pass Step II?
      1. How many attempts did it take you to pass?
      2. Score:
   3. Did you pass Step III?
      1. How many attempts did it take you to pass?
      2. Score:
7. Literature suggests that students who complete a gap year have lower rates of burnout when attending professional school. Do you agree with this statement? Y/N
   1. Optional comment:
8. What are the strengths of the gap year program?
9. What are opportunities for improvement of the gap year program?

Thank you for taking the time to complete this survey. Your feedback is valuable to us in enhancing the program for future participants and informing admission committees on applicant success.
