# Supplementary material for: Bridging the gap: evaluation of the impact of a structured pre-professional medical education gap year on medical career pathway, competency development, and preparedness for professional school
Source: BMC Med Educ. 2026 Jan 17;26:248. doi: 10.1186/s12909-025-08501-z (PMC12895797; doi:10.1186/s12909-025-08501-z)
Supplement: Supplementary file 2 — Supplementary Material 2. [file 12909_2025_8501_MOESM2_ESM.docx]

**Structured Clinical Gap Year Program Description**

**Participant Selection**

- **On-line Application**
- **Interview:**
  - 1^st^ Interview: In person (preferred) or by Zoom with program leadership (physician director, coordinator and administrator).
  - Optional 2^nd^ Look: job shadowing experience for clarity in expectations.
  - Program leadership assigns participant to provider based on mutual fit.

**Onboarding:**

- - HR standard process for all employees
  - Additional Gap Year training:
    - Patient care and history taking
    - Dermatologic techniques
    - Administering local anesthesia
    - Wound care
    - EHR documentation
    - Patient education
  - Completion of necessary certifications (e.g., Medication Aide, CITI training).

**Training**

- **Graded Responsibilities on a Timeline**
  - Months 1-3
    - Onboarding as above
    - 10-week Guided progression of clinical skill mastery (examples below)
      - Stock exam rooms
      - Autoclave and prepare instruments
      - Room patients
      - Document in EHR
      - Assist in simple then more complex procedures
      - Nursing duties
  - Months 3-4: Become a fully functioning team member
  - Months 6-12: Undertake “above and beyond” responsibilities
  - Months 12-14: Assist in the training of new gap year participants

**Comprehensive Clinical Experience:**

- Participate in all healthcare team activities
- Identify and promote of excellence in patient care
- Develop Clinical skills with progression based on competency

**Educational Curriculum**

- **Bi-Weekly Didactics:**
  - Delivered by providers.
  - Students are excused from other duties.
  - Students log hours and are compensated.
  - Scheduled lecture-based or interactive white board presentations.
  - Additional formal and informal sessions.
  - Ensure well-rounded and standardized education.
  - Enhance contributions to the healthcare team.
  - Preparation for future academic pursuits.
- **MCAT Content:**
  - In-depth reviews of life science subjects to reinforce fund of knowledge, critical analysis and develop test-taking strategies.
- **Pre-clinical and USMLE Content:**
  - In-depth reviews of key pre-clinical subjects develop a fund of knowledge and reinforce test-taking skills.
    - Anatomy
    - Immunology
    - Histology
    - Microbiology
    - Cancer Biology
    - Dermatology
- **Clinical Skills:**
  - Lectures and hands-on/simulation training on procedural techniques to assure standardized competency across gap year participants.
    - Sharps training
    - Aseptic and sterile technique
    - Suturing
    - Wound care
    - Injectable medication preparation
- **Job Performance Enhancement:**
  - Lectures on essential clinical skills needed for daily duties.
    - Local anesthetic administration
    - Surgical Assisting
- **Personal Development and Professional Success:**
  - Sessions developed to promote financial wellness and identify career pathways.
    - Financial literacy
    - Career planning

**Mentorship**

- **Emphasis on Strong Relationships:**
  - Close interaction with providers throughout various aspects of the program.
  - Continues throughout professional education and career.
- **Long-Term Interaction:**
  - Program allows providers to thoroughly assess and provide formative and summative feed-back on participants’ capabilities and longitudinal progression.
    - Job performance
    - Work ethic
    - Teamwork
    - Dedication
- **Letters of Recommendation**
  - Highly personalized letters of recommendation that accurately reflect attributes, ability and potential in chosen career.

**Research Opportunities**

- **Encouragement for Research Participation:**
  - Protocol development
- IRB submission
- Data collection
- Abstract or manuscript preparation and submission
- **Ongoing Projects Include:**
  - Original research
  - Program improvement
  - Retrospective chart reviews
  - Case reports
  - Best practices
- **Support for National Meetings:**
  - Based on degree of involvement, participants are financially supported to attend and present research at local and national meetings.
